# Spatial distribution and multilevel analysis of the ideal number of children among Ethiopian women

**DOI:** 10.1186/s12905-023-02477-y

**Published:** 2023-07-12

**Authors:** Addisalem Mengist, Demeke Lakew Workie, Zelalem G. Dessie, Lijalem Melie Tesfaw

**Affiliations:** 1 Department of Statistics, Debre Markos University, Amhara, Ethiopia; 2 Department of Statistics, Bahir Dar University, Bahir Dar, Amhara, Ethiopia; 3 School of Mathematics, Statistics and Computer Science, KwaZulu-Natal University, Durban, South Africa; 4 Epidemiology and Biostatistics Division , The University of Queensland, Brisbane, Australia

**Keywords:** INC, Women, Count data, Spatial analysis, Multilevel analysis, Ethiopia

## Abstract

**Background:**

Ideal number of children (INC) is the number of children that a woman or man would have if they could go back to the time when they did not have any children and could choose accurately the number of children to have in their total life. Despite numerous studies on the prevalence and associated factors of the ideal number of children, there is a lack of studies that incorporated spatial and multilevel analysis. Thus, this study was aimed at the spatial and multilevel analysis of an ideal number of children and associated factors.

**Methods:**

The study design was a cross-sectional study in which the data was obtained from Ethiopian Demographic and Health Survey (EDHS) in 2016. About 13,961 women ages 15–49 who fulfill the inclusion criterion were considered. A negative binomial regression model that incorporates spatial and multilevel analysis was employed.

**Results:**

About 33 and 12.8% of the women had four and six ideal numbers of children respectively. The highest INC per woman was recorded in Oromia region 5055 (36.1%) and the lowest in Harare 35(0.2%). The INC per woman is high in rural 10,726 (76.6%) areas as compared to urban areas 3277(23.4%). The ideal number of children was spatially clustered (Global Moran’s I = 0.1439, p < .00043). Significant hotspot clusters were found in the Somali region such as in Afder, Shabelle, Korahe, and Doolo zone.

**Conclusion:**

The spatial analysis revealed a significant clustering of the ideal number of children in the Ethiopia zone. Specifically, higher INC was observed in the Somali region, specifically in the Afder, Shabelle, Korahe, and Doolo zones. Among the various factors considered, women’s age, region, place of residence, women’s education level, contraception use, religion, marital status, family size, and age at first birth year were identified as significant predictors of the ideal number of children. These findings indicate that these factors play a crucial role in shaping reproductive preferences and decisions among women in the study population. Based on these findings, responsible bodies should prioritize targeted interventions and policies in high-risk regions to address women’s specific reproductive needs.

## Introduction

Fertility, as a fundamental component of population dynamics, plays a significant role in shaping the size and composition of a population over time. It refers to the ability and propensity of individuals or couples to bear children and is influenced by a multitude of factors, including social, economic, cultural, and health-related aspects. Changes in fertility patterns can have profound effects on population growth, age distribution, and demographic trends [1]. Fertility patterns in most of the created world within the late 1990s appeared a considerable decrease to two children or less from the conventional six children per woman [2]. In sub-Saharan Africa, fertility and projected population growth are much higher than in any other part of the world, and the already modest decline in fertility has slowed further in the last decade [3]. Among the sub-Saharan African countries, Ethiopia is one of the developing countries with high fertility and rapid population growth [4]. According to EDHS, the birth rate in Ethiopia experienced a slight decline between 2000 and 2005, decreasing from 5.5 to 5.4 children per woman. Subsequently, the birth rate further to 4.8 children per woman in 2011 and reached 4.6 children per woman in 2016. This indicates a general decline in the total fertility rate (TFR) in Ethiopia over time. The TFR decreased by 0.9 children, from 5.5 children per woman in 2000 to 4.6 children per woman in 2016. Notably, the most significant decline occurred between the two most recent 5-year periods, specifically in 2016 [5].

In rural areas, parents want to have many children to have support in farming and emotional and economic support in old age [6]. According to the 2016 EDHS report, fertility is lower in urban Ethiopia than in rural Ethiopia. Like other creating countries, significant variation in fertility level was watched among rural and urban inhabitants of Ethiopia. There are clear regional contrasts in fertility levels and patterns in Ethiopia [7]. Some of the regions like Addis Ababa have achieved a below replacement level of fertility (TFR = 1.8) while others such as Oromia (5.4) and Somali (7.2) regions have total fertility rates that are above the national average [8].

The ideal number of children represents the hypothetical number of children that individuals would choose to have if they had the opportunity to make a fully informed decision about their family size before having any children [7, 8]. This concept recognizes that individuals may have different preferences and aspirations regarding the number of children they would like to have throughout their lifetime. Research has demonstrated that the actual number of children individuals has influenced their desired number of reported children [8].

The study done by [9] on the desired number of children (DNC) among women in Ethiopia, using a count regression model, highlights the need for in-depth research on various aspects related to the DNC. Therefore, the present study aims to identify the risk factors associated with the desired number of children in different regions of Ethiopia, considering the diverse socio-economic, demographic, health, environmental, and sociocultural factors that influence DNC among Ethiopian women. This study seeks to address regional variations and practices related to the DNC, while exploring the factors that influence women’s DNC decisions, considering various health, socio-economic, and environmental factors such as women’s age, education, place of residence, and economic status of the household, among others. This study would contribute to improving access to reproductive healthcare, promoting contraception education, and addressing social and cultural factors influencing reproductive decisions. Moreover, it would help empower women and provide support, enabling informed choices regarding ideal family size and leading to improved reproductive health outcomes in Ethiopia.

## Conceptual framework

**Fig. 1:**
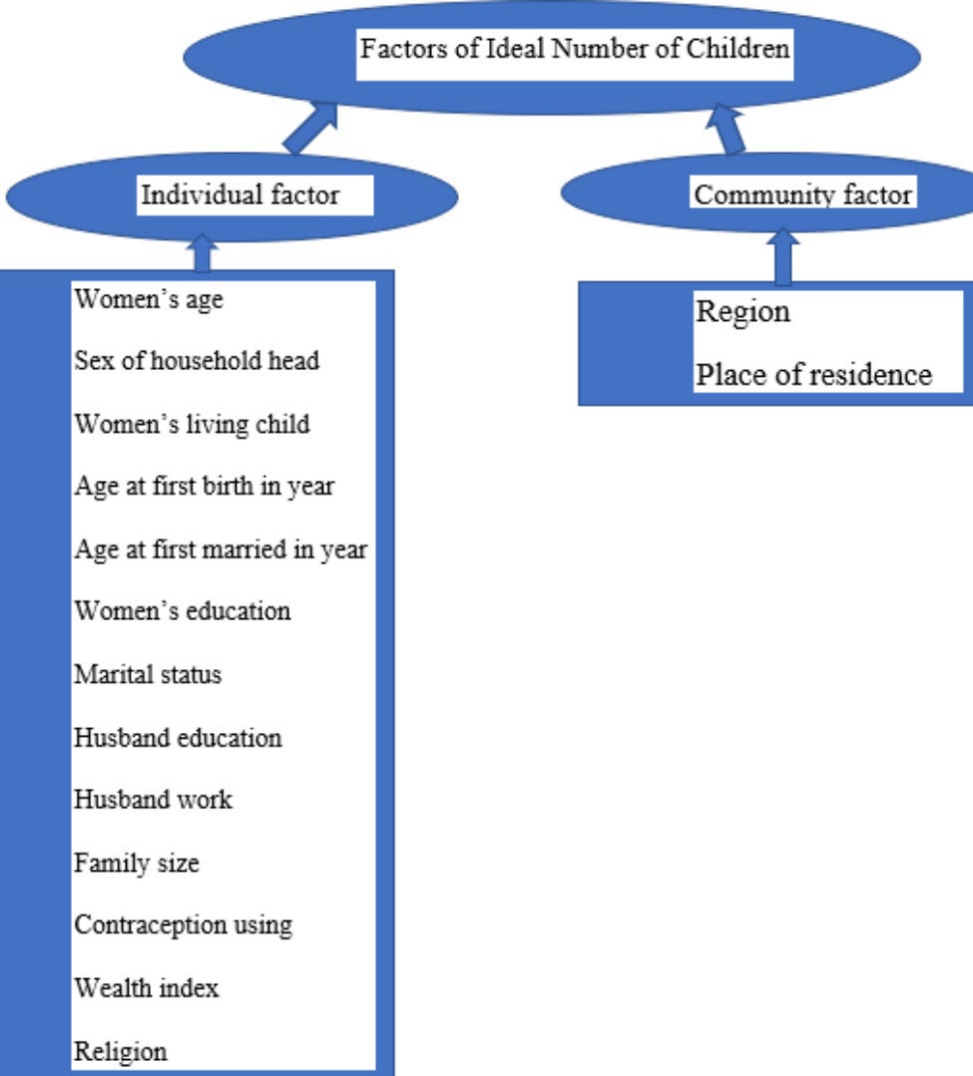
Conceptual framework for factors of INC

The ideal number of children (INC) has been explained by different theories like the social and economic, and public health explanation [3]. INC differences among populations and trends in desired over time can always be traced to variations in one or more of the intermediate ideal variables. Figure 1 summarizes the relationships among the determinants of INC.

## Methods

### Study area and design of the study

Ethiopia is a country in the Horn of Africa. The country lies completely within the tropical latitudes and is relatively compact, with similar north-south and east-west dimensions. Administratively, Ethiopia is federally decentralized into eleven regions, and two city administrations. Divided into 74 zones with 817 administrative units called districts. Ethiopia is the largest and the second most populous country in Africa with a high fertility rate of 4.6 children per woman [8].

The 2016 EDHS employed a cross-sectional study design. The EDHS survey was designed to provide nationally representative data on various demographic and health indicators in Ethiopia. The survey utilized a stratified two-stage cluster sampling approach. In the first stage, enumeration areas (EAs) were selected from a sampling frame based on the 2007 Ethiopian Population and Housing Census. In the second stage, a fixed number of households were selected from each selected EA using systematic random sampling [4, 8].

### Data source and population

The data for this analysis was obtained from the 2016 EDHS. The EDHS is a survey designed to provide population and health indicators at the national and regional levels [10]. The CSA has surveyed in collaboration with the Federal Ministry of Health (FMoH) and the Ethiopian Public Health Institute (EPHI) with technical assistance from ICF International, and financial as well as technical support from development partners [8]. The study population for this study included all women aged 15–49 years across all regions of Ethiopia. About 18,008 households were selected using a two-stage stratified cluster sampling method of which 17,067 were occupied. Among 15,683 women who were interviewed, these studies consider 13,961 women having a numeric response for the INC.

### Variables include in the study

#### Response variable

The response variable of this study is the ideal number of children that a woman would like to have during their reproductive age (15–49). This dataset has one record for every eligible woman as defined by the household schedule. It contains all the data collected in the woman’s questionnaire plus some variables from the household.

#### Predictor variables

Possible potential predictors of the ideal number of children was presented in Table 1.

**Table 1:** Classification of the independent variables

Predictor variables		Category	
Region		1 = Tigray, 2 = Afar, 3 = Amhara, 4 = Oromia,, 5 = Somali, 6 = Benshangul G.,7 = SNNPE, 8 = Gambela, 9 = Harari, 10 = Addis Abeba and 11 = Dire Dew	
Woman’s age		1 = 15–19, 2 = 20–24, 3 = 25–29, 4 = 30–34, 5 = 35–39, 6 = 40–44 and 7 = 45–49	
Sex of household head		1 = Male,2 = Female	
Women’s living child		1 = no, 2 = yes	
Contraception using		1 = no,2 = yes	
Place of residence		1 = urban, 2 = rural	
Wealth index		1 = poorest, 2 = poorer, 3 = middle, 4 = richer and 5 = richest	
Women’s work		1 = no employed, 2 = employed	
Women’s education		1 = no education, 2 = primary,3 = secondary and 4 = higher	
Religion		1 = Orthodox, 2 = Catholic, 3 = Protestant, 4 = Muslim, 5 = traditional and 6 = other	
Marital status		1 = single, 2 = marriage, 3 = Separated/Divorced/Widowed	
Husband education		1 = no education,2 = primary,3 = secondary,4 = higher,5 = don’t know	
Husband work		1 = no employed and 2 = employed	
Family size		Count	
Age at first married (yrs)		Continuous	
Age at first birth (yrs)	Continuous		

**Key**: SNNPR = South Nations Nationalities and Peoples Region

### Statistical methods

#### Count regression models

Given the nature of our outcome of variable, the ideal number of children, it is more suitable to employ count regression models such as Poisson or negative binomial regression. Poisson regression assumes equal variance and means of the outcome variable [11]. However, in cases where this assumption is not met, the negative binomial regression model is a better alternative. In our study, where the variance exceeded the mean, we utilized a negative binomial regression model with multilevel and spatial analysis.

#### Multilevel count regression models

Multilevel modeling is a data analysis method that is frequently used to investigate hierarchical data structures. Multilevel data analysis exploits data structures that cannot be adequately investigated using single-level analytic methods. Hence, multilevel models have become increasingly popular in analyzing data organized in nested or repeated measurements. Explaining variability in a multilevel structure can be achieved by explaining variability between level-1 units and also by explaining variability between higher-level units [5]. In this study, we have a hierarchy in which the ideal number of children in a region is expected to be correlated. This was checked using intra-class correlation (ICC). ICC quantifies the proportion of the variance explained by the region in the population and simply states that the ICC is the proportion of region-level variance compared to the total variance. It can be calculated as:

3.24 $${\bf{ICC}} = {{\bf{\delta }}^2}_{{\rm{uo}}}/({{\bf{\delta }}^2}_{{\rm{uo}}} + {{\bf{\delta }}^2}_{\rm{e}})$$

Where, ${{\delta }}_{\text{e}}^{2}$the variance of the individual is (lower) level units and ${{\delta }}_{\text{u}\text{o}}^{2}$ is the variance of the higher-level residual errors. In the multilevel logit model, level-one residual variance ${{\delta }}_{\text{e}}^{2} = {\pi ^{2/3}} \approx 3.29$ [12]. Therefore, the above Eq. (3.24) can be reformulated as:

$${\bf ICC }= {\varvec{\delta }}_{\mathbf{u}\mathbf{o}}^{2} / \left({\varvec{\delta }}_{\mathbf{u}\mathbf{o}}^{\bf 2}+ {\bf 3.29 }\right)$$

#### Spatial analysis

Spatial analysis is an analysis, which includes the influence of spatial or space in the analysis. In spatial analysis, there is always a correlation between spaces, which is called spatial correlation [13]. In this study, the spatial autocorrelation between ideal numbers of children across [13]zones was considered. The spatial autocorrelation was computed using a weighted matrix [14].

The weighted matrix is used to evaluate spatial autocorrelation. First, identify neighbor zones that share common boundaries and choose the distance measure [14]. These distances are presented in a weight matrix (Wij), which defines sometimes the so-called Contiguity matrix that describes the relationship between districts i and j in the specified area. The (i, j) the element of a spatial proximity matrix W, denoted w_ij_, quantifies the spatial dependence between regions i and j, and collectively, the w_ij_ define a neighborhood structure over the entire area. The spatial correlation parameter and W=(w_ij_) is a neighborhood matrix for the areal units [15]. Which, can be defined as:

$${\text{w}}_{\text{i}\text{j}}=\left\{\begin{array}{c}1, if\, districts i\, and\, j\, share\, a\, common\, boundary ij\\ 0, otherwise \end{array}\right.$$

Moran’s I: Moran’s I [16] tests for global spatial autocorrelation or the correlation of a variable with itself across space. It is based on the cross-products of deviations of observations from their mean and is calculated by accounting for the location of the observations. Moran’s I calculated for n observations on a variable “x” at locations *i*, j can be written as follows:

$$\text{I}= \frac{\text{n}}{\text{S}\text{o}}\frac{\sum _{\text{i}=1}^{\text{n}}\sum _{\text{i}=1}^{\text{n}}\text{w}\text{i}\text{j}(\text{x}\text{i}-\stackrel{-}{\text{x}})(\text{x}\text{j}-\stackrel{-}{\text{x}})}{\sum _{\text{i}=1}^{\text{n}}(\text{x}\text{i}-\stackrel{-}{\text{x}})2}$$

Where n is the number of polygons (clusters in this study) or several observations in the variable x. $\stackrel{-}{\text{x}}$ is the mean of the x, ${\text{w}}_{\text{i}\text{j}}$ are the elements of the weight matrix, and *S*_0_ is the sum of the elements of the weight matrix:

$${\text{s}}_{0}=\sum _{\text{i}=1}^{\text{n}}\sum _{\text{j}=1}^{\text{n}}{\text{w}}_{\text{i}\text{j}}$$

the spatial weighted matrix (wij) is written as:

$$\left[\begin{array}{ccc}\text{w}11& \cdots & \text{w}1\text{n}\\ \vdots & \ddots & \vdots \\ \text{w}\text{n}1& \cdots & \text{w}\text{n}\text{n}\end{array}\right]$$

Moran’s I is varying from − 1 to + 1. Moran’s I values close to − 1 indicate spatial distribution is dispersed, whereas Moran’s I values close to + 1 indicate spatial distribution is clustered, and Moran’s I value of 0 means distributed randomly.

Local Getis-Ord Gi* statistics were used to identify the hot and cold spot areas using GPS latitude and longitude coordinate readings that were taken at the nearest community center for zones [16]. The Ordinary Kriging geostatistical interpolation method was used to predict INC in unobserved areas of the Ethiopia zone.

### Multilevel model with spatial auto covariate structure

The spatial process was modeled with the normal conditional autoregressive random effect which can incorporate the multilevel effects. The multilevel modeling approach has a convenient way to deal with spatial autocorrelations by using a spatial auto covariate model [17]. We extend the random coefficient model to a spatial model by adding an auto-covariate structure:

$$\begin{aligned}{\rm{log}}({\mu _{ij}}) & = {\beta _0} + {\sum\nolimits_{{\bf{(i = 1)}}}^{\bf{k}} \beta _{\bf{i}}}{{\bf{x}}_{{\bf{lij}}}} + {\mu _{oj}} + \rho {{\rm{s}}_{\rm{i}}} \end{aligned}$$

Where ρ is a parameter to be estimated that determines the direction and magnitude of the spatial neighborhood effect, s_*i*_ is calculated as:

$${\mathbf{s}}_{\mathbf{i}}=\frac{\sum _{\mathbf{j}=1}^{\mathbf{n}\mathbf{i}}{\mathbf{w}}_{\mathbf{i}\mathbf{j} }{\mathbf{y}}_{\mathbf{i}} }{\sum _{\mathbf{j}=1}^{\mathbf{n}\mathbf{i}}{\mathbf{w}}_{\mathbf{i}\mathbf{j}}}$$

*w*_*ij*_ is spatial weighting matrices. The auto covariate variable (S_i_) is a weighted average of the geographic units among a set of n_i_ neighbors of the geographic unit i. Where *y*_*j*_ is the response value of y at site j among the site i’s set of n_i_ neighbors; the spatial weight between the geographic unit i and j is ${\text{w}}_{\text{i}\text{j}}$.

## Results

Table 2 below shows that 1,298(9.3%) women do not need any ideal number of children whereas 112(0.8%) women need only one INC and 4,607(33%) women need only four ideal number of children.

**Table 2:** Weighted frequency table of the ideal number of children

Ideal number of children	Frequency	Percent
0	1298	9.3
1	112	0.8
2	1592	11.4
3	1145	8.2
4	4607	33.0
5	1243	8.9
6	1787	12.8
7	433	3.1
8	754	5.4
9	70	0.5
10	614	4.4
11	14	0.1
12	168	1.2
13	14	0.1
14	14	0.1
15	56	0.4
Total	13,961	100

### Summary of predictors related to the INC among Ethiopia women

Some of the socioeconomic, demographic, health, and environmental related factors on the ideal number of children were presented in Table 3. The highest INC per woman was recorded in Oromia region 5055 (36.1%) and the lowest in Harare 35(0.2%). The INC per woman is high in rural 10,726 (76.6%) areas as compared to urban areas 3277(23.4%). According to religion, the INC per woman is lowest at 77(0.5%) for women of the catholic religion, and the highest INC per woman occurred for women’s Orthodox religion (43.5%). According to the age of women, among the age groups of women, the INC per woman is high (22.8%) among the youngest age group (15–19) and small 820(5.9%) in the oldest age group (45–49). Suggesting that INC per woman was negatively related to the age of women.

According to the sex of the household head, the INC male household head of 10,676 (76.2%) was greater than that of female 3326 (23.8%). In the same way, women with no work had the highest INC per woman as compared to women that had work. According to contraception use, the INC per woman among contraceptive users 10,391 (74.2%) was higher than that of women’s not used contraceptive methods 3612(25.8%). The average family size, age at first marriage in the year, and age at first birth in the year for the INC was 5.51, 17.1, and 18.83 respectively, etc.

**Table 3:** Summary Statistics of Predictor Variables related to INC in Ethiopia, (EDHS-2016)

Variables	Category	frequency	Percent
Region	Tigray	967	6.9
	Afar	102	0.7
	Amhara	3278	23.4
	Oromia	5055	36.1
	Somali	347	2.5
	Benishangul	145	1.0
	SNNPR	3040	21.7
	Gambela	41	0.3
	Harari	35	0.2
	Addis Adaba	910	6.5
	Dire Dawa	82	0.6
Woman’s age	15–19	3187	22.8
	20–24	2589	18.5
	25–29	2654	19.0
	30–34	2023	14.4
	35–39	1667	11.9
	40–44	1062	7.6
	45–49	820	5.9
Place of residence	Urban	3277	23.4
	Rural	10,726	76.6
Sex of household head	Male	10,676	76.2
	Female	3326	23.8
Wealth index	Poorest	2183	15.6
	Poorer	2462	17.6
	Middle	2671	19.1
	Richer	2777	19.8
	Richest	3910	27.9
women’s working	No	9290	66.3
	Yes	4713	33.7
Women’s educational level	No education	6303	45.0
	Primary	5093	36.4
	Secondary	1746	12.5
	Higher	860	6.1
Current marital status	Single	3843	27.4
	Married	8716	62.2
	Separated	1443	10.3
Religion	Orthodox	6098	43.5
	Catholic	106	0.8
	Protestant	3422	24.4
	Muslim	4204	30.0
	Traditional	96	0.7
	Other	77	0.5
Current contraceptive using	Not using	10,391	74.2
	Using	3612	25.8
Women have living children	Yes	4904	35.0
	No	9099	65.0
Husband’s education level	No education	3924	28.0
	Primary	3338	23.8
	Secondary	902	6.4
	Higher	677	4.8
	Don’t know	58	0.4
Husband’s working	No	655	4.7
	Yes	8149	58.2
	Don’t know	94	0.7
Continuous predictors		Mean	St.dev
Family size	Count	5.51	2.408
Age at marring in year	Continuous	17.11	3.933
Age at first birth in the year	Continuous	18.83	3.630

### Spatial analysis of ideal number of children

**Fig. 2:**
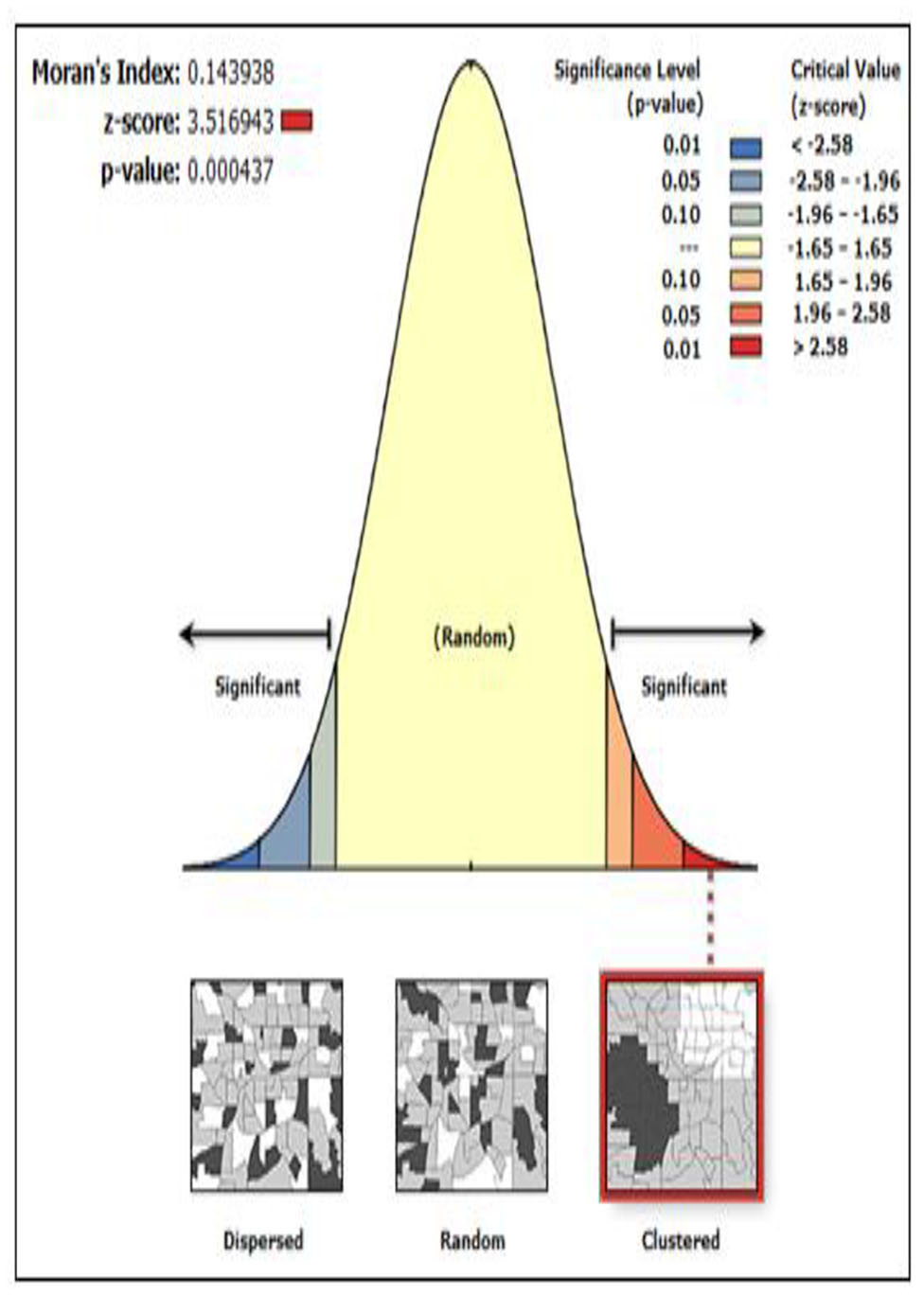
Spatial autocorrelation for distribution of the ideal number of children, (EDHS-2016)

Moran’s I index measures the spatial autocorrelation, in the sense that the index allows detecting whether or not INC in a given cluster is similar or not to that of neighboring zones. The outputs have automatically generated keys on the right and left sides of the panel. The bright red and blue colors (to the end tails) indicate an increased significance level. It evaluates whether the pattern expressed is clustered, dispersed, and random or tests the hypothesis of the absence of spatial autocorrelation for the response variable. For this study, the estimated Global Moran’s Index value of an ideal number of children was 0.1439; When the Moran’s I values near 1 showed that the event was clustered therefore the values Moran’s I is almost near one this indicates that the INC are clustered in Ethiopia. In addition to looking at the Moran’s I to identify whether there is a spatial correlation, the P-value ( 0.00043) was found to be less than 0.05, suggesting significant evidence of spatial autocorrelation in INC (Table 4; Fig. 2). Similarly, the incremental spatial autocorrelation graph identified the maximum peak distance value, which indicates distances where spatial processes promoting clustering are most pronounced. The color of each point on the graph corresponds to the statistical significance of the z-score values.

**Table 4:** Moran’s index statistic for spatial autocorrelation

Moran’s Index:	Variance:	z-score	p-value:
0.143938	0.002009	3.516943	0.000437

### Spatial distribution of Ideal Number of Children

**Fig. 3:**
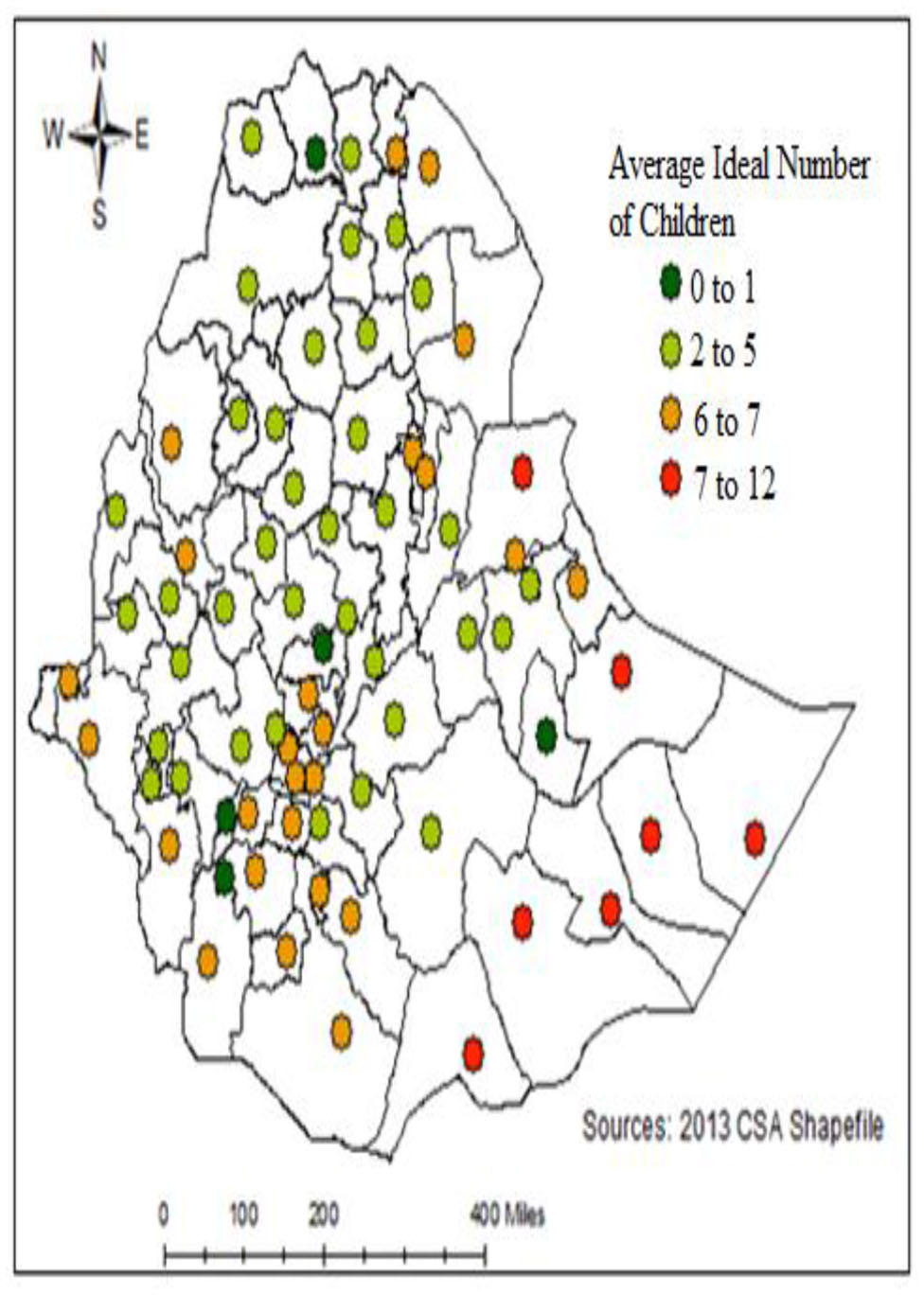
Spatial distribution of the ideal number of children in Ethiopia, (EDHS-2016)

The spatial distributions of the ideal number of children, each point in the map characterizes the enumerations area with the desired or ideal in each zone of Ethiopia (see Fig. 3). The red color indicates areas with a high average ideal number of children and the area green color indicates a low average ideal number of children.

### Cluster and outlier analysis of ideal number of children

Cluster and outlier analysis was conducted to identify the nature of clustering by using Anselin.

**Fig. 4:**
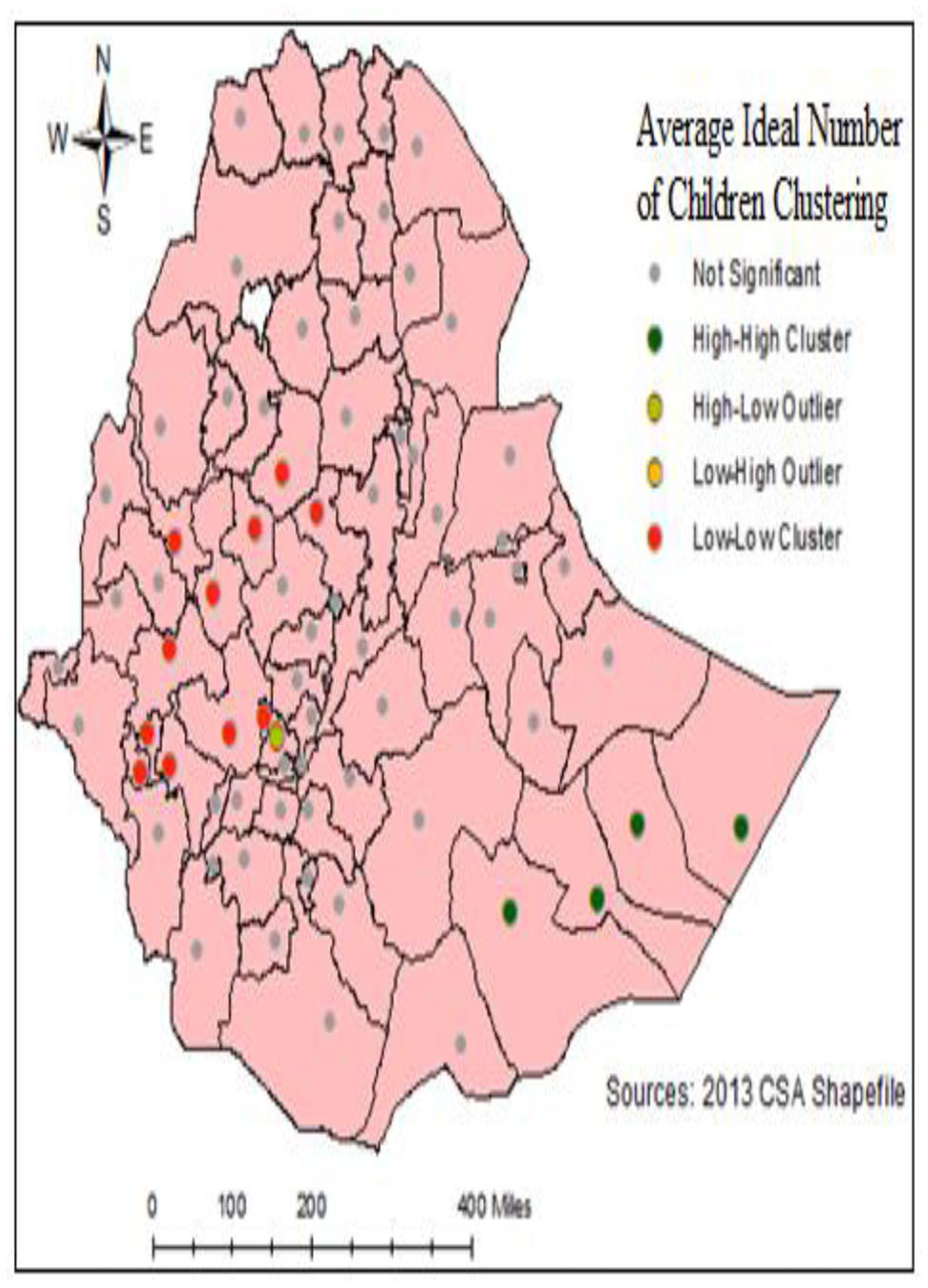
Spatial Clustering of INC in Ethiopia women based on the Local Moran’s I

Local Moran’s I. A positive value for Local Moran’s I indicate that a feature has neighboring features with similarly high or low attribute values this feature is part of a cluster. A negative value for Local Moran’s I indicate that a feature has neighboring features with dissimilar values this feature is an outlier and the P-value must be small for the cluster or outlier to be considered statistically significant. In the Somali region in Afder, Shabelle, Korahe and Doolo are some of the zones that are high Clustering of the ideal number of children, see Fig. 4.

### Hot spot analysis of ideal number of children

**Fig. 5:**
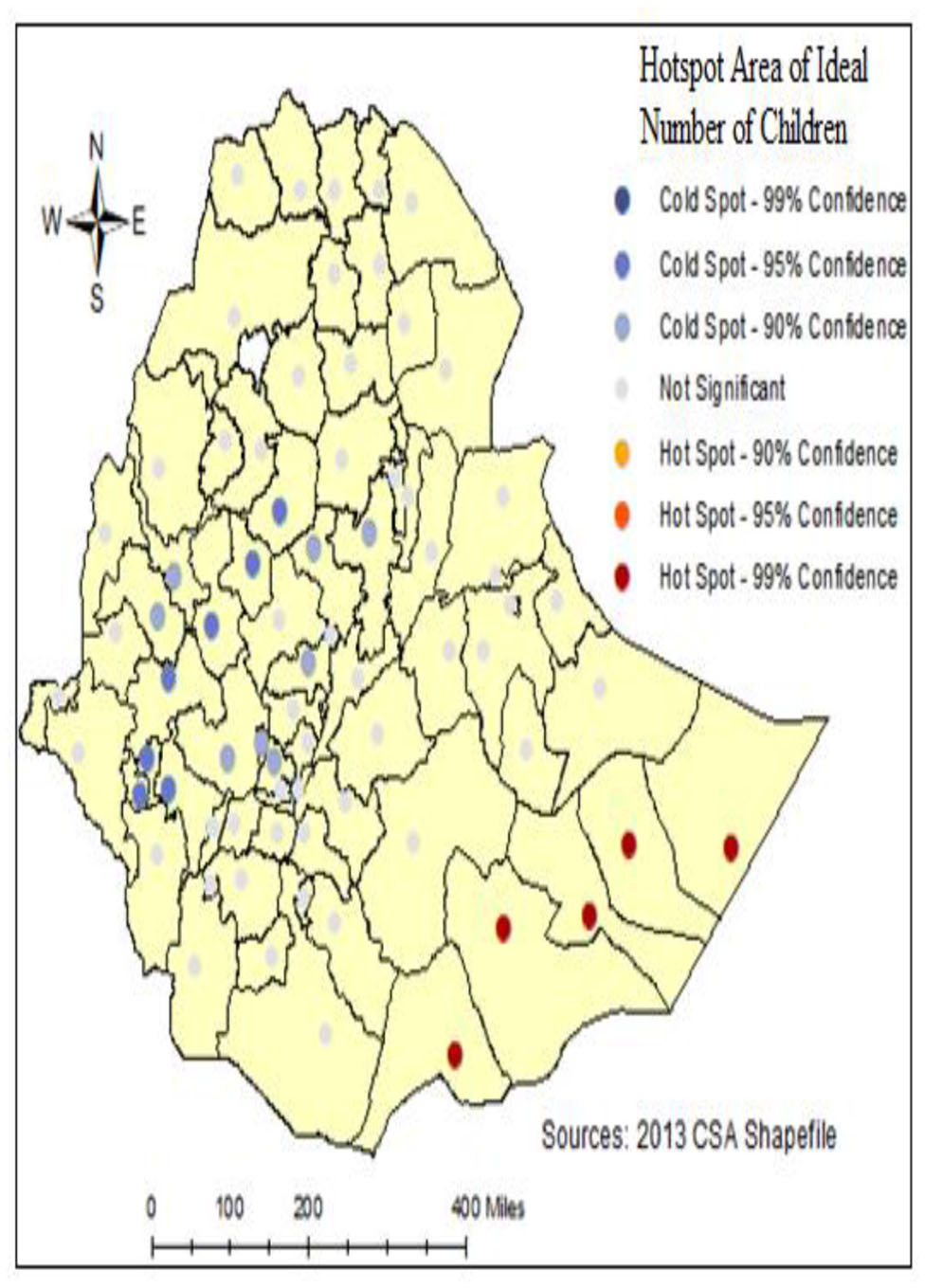
Hotspot analysis of INC across regions in Ethiopia, (EDHS- 2016)

Hotspot analysis uses to identify the locations of statistically significant hot spots and cold spots in the data. The blue color indicates the significant cold spot (low-desired) areas of several children per zone and the red color indicates the hotspot (high-desired) areas of many children. For example, there is a high Somali region in Afder, Shabelle, Korahe, and Doolo zone (see Fig. 5).

### Spatial interpolation of ideal number of children

**Fig. 6:**
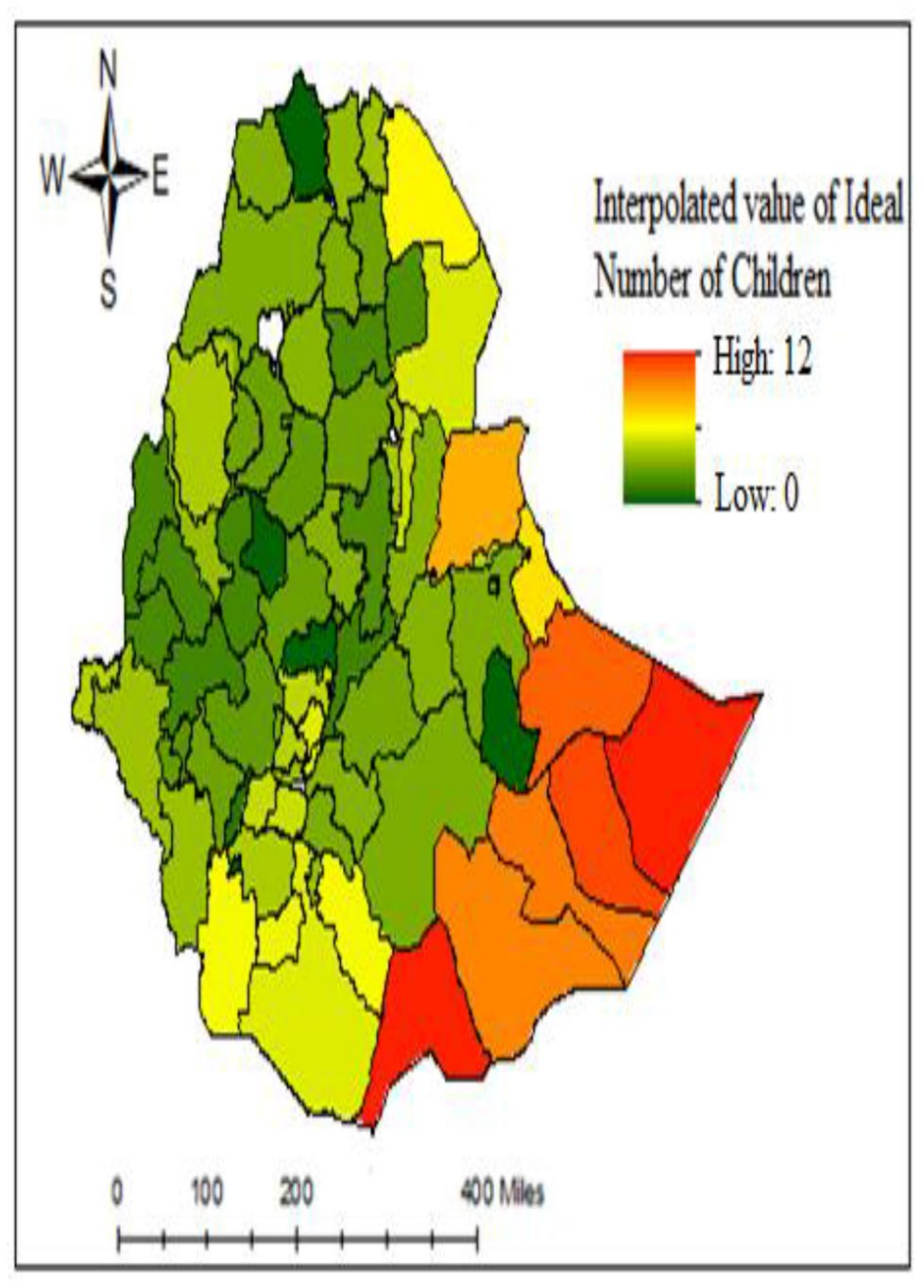
Spatial interpolation of INC among women in Ethiopia zone (EDHS-2016).

Ordinary kriging interpolation to predict ideal children status for unknown areas of the country based on sampled enumeration area measurements was presented in Fig. 6. The area indicated by red color indicates the higher average ideal number of children.

### Multilevel count regression analysis of the data

In this analysis, we expected that there would be a difference in the INC from individual women among Ethiopia zones. We did this considering the zone variation of women’s place of living as one factor for the INC. We consider multilevel models to allow and explore the between-zone variance of INC. The data have a two-level hierarchical structure with 13,928 women’s at level 1, nested within 74 zones at level 2.

### Test of heterogeneity

For the proper application of multilevel analysis, first, we have to test for heterogeneity of an ideal number of children among zone. The likelihood ratio test was applied to assess heterogeneity among zones. The test yields, LRT = 400.604 at P-value = 0.001which is significant at 5%, implying strong evidence of heterogeneity for an ideal number of children in Ethiopia zones. Therefore, multilevel analysis is better.

### Spatial multilevel NB regression with autocovariate models

The results of fitting the multilevel models of an ideal number of children. Table 5 shows that in the random effect for NB regression, estimates for intercepts and the slopes vary significantly at a 5% significance level, which infers that there is a significant variation in the effects of family size these variables differ significantly across the zone. The value of 0.00414 and 0.000208 is the estimated variance of intercept (zone) and family size respectively.

Results of random coefficients with auto covariate model, an Autocovariate variable is a spatial neighborhood effect variable that addresses spatial autocorrelation by estimating the mean ideal number of children of exp(0.0345) one more likely the ideal number of children at any one zone reflects an ideal number of children at surrounding zones. There is a positive spatial neighborhood effect (0.0345). The p-value is 0.007 also proves that it was true, in the sense that there was spatial autocorrelation of an ideal number of children between zones. The spatial neighborhood effect with the ideal number was a positive value, 0.0345 indicating that there is a Positive spatial Autocorrelation between zones with similar values occurring in a similar zone when other variables held constant.

Predictors statistically significant in the count model are women’s age, women’s education, region, place of residence, Contraception use, religion, marital status, family size, and age at first birth in the year were statistically significant. The estimated coefficients for all age groups are positive and show that the INC increases with the incremental age of women. The expected INC that women aged 25–29 would like to have in their lifetime was 8.1% (IRR = 1.081183) times higher than women aged 15–19 would like to have in their lifetime. The expected INC that women aged 30–34 were 12.2% (IRR = 1.122461) times higher than women aged 15–19 would like to have to control all other variables in the model and random effect at level two.

The findings of this study also showed that Region had a significant effect on the INC per woman. The expected INC region (Amhara, Oromia, SNNPR, and Addis Ababa) were decreased by 83.4%(0.8344664), 76.4%(0.7645548), 91%(0.9106741) and 82.7%(0.8273197) as compared with the Tigray region respectively and the expected INC region in Somali was increased by72.2% (1.721917) as compared with the Tigray region. The findings of this study also revealed that women’s education has a significant effect on the INC. The expected INC for women with primary, secondary, and higher education were 93.5% (IRR = 0.9350458 89.1% (0.8918267), and 89.6%(IRR = 0.8966436) respectively times lower as compared to those with non-educated. Likewise, the negative and statistically significant coefficient, -0.08291 suggest that the expected log of INC that women using contraceptives would like to need in their lifetime is 8.2% less than women not using a contraceptive.

The coefficients for the categories of marital: separation was positive and statistically significant, implying that the expected INC was 4.6% (IRR = 1.04617) respectively times the higher need for INC than single women. The estimated coefficient for age at first birth was negative and statistically significant, implying that as age at first birth increased by one year the expected INC decreased by 98.8%(IRR = 0.9888356).

**Table 5:** The results of random coefficient with auto covariate predictor NB regression model

Parameter	Coef.	IRR	Std. Err.	Z-value	P-value	[95% CI for IRR] Lower Upper	
Intercept	1.404	4.072	0.436	13.13	0.000	3.300	20.070
Age(15–19)(Ref.)							
20–24	0.021	1.021	0.018	1.220	0.223	0.987	1.056
25–29	0.078	1.081	0.020	4.240	0.000	1.043	1.121
30–34	0.116	1.122	0.023	5.750	0.000	1.079	1.168
35–39	0.209	1.232	0.025	10.110	0.000	1.183	1.283
40–44	0.265	1.303	0.029	11.780	0.000	1.247	1.362
45–49	0.281	1.323	0.032	11.690	0.000	1.263	1.388
Place(urban)(Ref.)							
Rural	0.057	1.058	0.029	2.070	0.039	1.003	1.116
Region(Tigray)(Ref.)							
Afar	-0.052	0.949	0.065	-0.760	0.446	0.830	1.086
Amhara	-0.181	0.834	0.023	-6.430	0.000	0.790	0.887
Oromia	-0.268	0.766	0.023	-9.090	0.000	0.722	0.810
Somali	0.543	1.722	0.071	13.100	0.000	1.587	1.868
Benishan	-0.011	0.989	0.058	-0.180	0.855	0.882	1.109
SNNPR	-0.094	0.911	0.029	-2.960	0.003	0.856	0.969
Gambela	-0.084	0.920	0.020	-0.770	0.440	0.743	1.136
Harari	-0.201	0.818	0.103	-1.590	0.112	0.640	1.048
Addis Ababa	-0.190	0.827	0.037	-4.220	0.000	0.758	0.903
Dire Daw	0.060	1.062	0.088	0.730	0.468	0.903	1.249
Sex of Household head(male)(Ref.)							
Female	0.006	1.006	0.019	0.330	0.738	1.045	1.055
Women Work(No)(Ref.)							
Yes	-0.023	0.978	0.013	-1.690	0.091	0.952	1.004
Wealth index(poorest)(Ref.)							
Poorer	0.001	1.000	0.019	0.020	0.982	0.963	1.038
Middle	-0.027	0.973	0.019	-1.390	0.164	0.937	1.011
Richer	-0.021	0.979	0.020	-1.040	0.298	0.941	1.019
Richest	-0.003	0.997	0.026	-0.130	0.894	0.947	1.049
Women’s Education( No education)(Ref.)							
Primary	-0.067	0.935	0.014	-4.540	0.000	0.908	0.963
Secondary	-0.114	0.892	0.027	3.770	0.000	0.840	0.946
Higher	-0.109	0.897	0.035	2.810	0.005	0.831	0.968
Marital(single)(Ref.)							
Married	0.082	1.086	0.059	1.500	0.133	1.208	1.086
Separated	0.045	1.046	0.022	2.130	0.033	1.004	1.090
Family size	0.040	1.041	0.013	3.160	0.002	1 0.015	1.068
Religion(orthodox)(Ref.)							
Catholic	-0.117	0.890	0.070	-1.480	0.140	0.762	1.039
Protestant	0.057	1.059	0.022	2.770	0.006	1.017	1.103
Muslim	0.089	1.093	0.020	4.870	0.000	1.055	1.133
Traditional	0.091	1.096	0.076	1.310	0.189	0.956	1.256
Other	0.312	1.366	0.084	5.040	0.000	1.210	1.541
Contraception(Not using)(Ref.)							
Using	-0.083	0.920	0.012	-6.350	0.000	0.897	0.944
Husband’s work(No)(Ref.)							
Yes	-0.001	0.999	0.022	-0.060	0.950	0.956	1.043
Don’t know	0.0146606	1.015	0.069	0.220	0.829	0.888	1.159
Age at first birth (yrs)	-0.011	0.989	0.002	6.380	0.000	0.985	0.992
spatial auto covariate (Si)	0.035	1.035	0.027	1.320	0.007	1.003	1.090
Family size	-0.007	0.993	0.004	-1.670	0.004	0.984	0.998
α	2.760	-2.760	0.003			-2.903	-2.616
Zone	Variance	Rate ratio Std Dev.		95%confidenc interval			
${{\delta }}_{\text{u}0}^{2}$ (_cons)	0.004	0.004	0.002			0.0014	0.012
${{\delta }}_{\text{u}1}^{2}$ (Family size)	0.0002	0.0002	0.00007			0.0001	0.0004

key: Ref = reference, Std. Err. = Standard error, IRR = Incidence Rate Ratio, and α is dispersion parameter value < 0.05

## Discussion

This paper has demonstrated the ideal numbers of children in Ethiopia women and determined the factors that affect women’s status by considering INC from EDHS data. The study was evaluated based on different statistical models such as the NB regression model, random coefficient with auto covariate predictors, and spatial distribution. In the meantime, a model with a good fit of the data was identified by comparing the candidate model using deviance, AIC, and BIC. Of those models, the random coefficient with auto covariate predictors’ model was the relatively best model that manifests the data in the study, and hence, the effect of INC characteristics including the spatial effect on the women’s status was estimated using spatial.

The spatial analysis conducted in this study employed a global test for spatial autocorrelation to examine the clustering pattern of the ideal number of children across Ethiopia. The analysis revealed a significant spatial autocorrelation, indicated by a Moran’s I value of 0.1439 and a p-value < 0.001. This finding suggests that the spatial distribution of INC exhibits a notable clustering tendency within the Ethiopia zone. The observed spatial clustering finding is consistent with the results of previous studies conducted both within and outside Ethiopia, providing additional support for the presence of spatial clustering in the distribution of INC [2, 18]. Further examination of the research areas identified hotspots of INC IN THE Southeast and Eastern regions of the country. These specific counties demonstrated higher concentrations of INC compared to surrounding areas, indicating localized areas of increased fertility preferences [2]. The results of the Getis-Ord Gi* statistic supported and confirmed the results of the findings of the spatial analysis. In the Somali region: Afder, Shabelle, Korahe, and Doolo zone are identified as INC hotspots. Therefore, Somali was predicted to have more INC compared to other region zones. This finding can indicate that the clustering located in the Eastern parts of the country zone is a hotspot area [19].

There was not any pragmatic variable significant difference between fitting random intercept with auto covariate and random coefficient with auto covariate but there was a bit of deviance, AIC, and BIC difference and since a model with the smallest deviance, AIC, and BIC are preferred so random coefficient with auto covariate predicts was preferred.

The study findings demonstrated that the place of residence had a significant impact on the ideal number of children among women in Ethiopia. Specifically, the results indicated that women residing in rural areas had a higher mean number of INC compared to those residing in urban areas. This observation was consistent with similar findings reported by a previous study [20, 21]. The region is one of the significant important predictors of the INC. That is INC less in Amhara, Oromia, SNNPR, and Addis Ababa regions as compared to the Tigray region. Whereas, the Somali region is higher INC than the Tigray region. This finding is confirmed by [21, 22]. This might be because of several reasons. Variations in access to reproductive health services, including family planning and contraception, may exist between regions. Limited availability and awareness of family planning methods can result in a higher ideal number of children among individuals in the Somali region. Gender Roles and Empowerment: Gender roles and the status of women in society can influence reproductive decision-making. Factors such as gender inequality, limited decision-making power for women, and lack of empowerment opportunities may contribute to a higher ideal number of children in the Somali region. It is important to note that these justifications are speculative and would require further research and analysis to confirm their validity. Conducting in-depth studies and surveys within the Somali region, including qualitative research and interviews, can provide a better understanding of the specific factors contributing to the higher ideal number of children in that region.

The finding of the study revealed that the average INC for women aged (25–29, 30–34, 35–39, 40–44, and 45–49) is significantly higher than that of younger women. This finding is supported by [23]. A similar result was observed in another study done by [23, 24]. The study findings indicated that women’s level of education had a significant influence on the average number of ideal children. Women with higher levels of education had a decreased average ideal number of children compared to women with no education. This finding suggests that education plays a role in shaping women’s reproductive preferences and decisions. This result aligns with a previous study conducted by [3], which also reported a similar relationship between women’s education and INC. A study in [25] demonstrated that women’s educational attainment increase and there is a tendency for them to desire and aim for a smaller number of children. The impact of education on fertility preferences can be attributed to various factors. Education empowers women by providing knowledge, skills, and opportunities for personal and professional development. Educated women are more likely to have access to information and resources related to reproductive health, contraception, and family planning. They may also have a greater awareness of the social, economic, and health implications of having a large family, leading to a desire for smaller family sizes. The consistency of these findings across multiple studies, including the present study and [5], highlights the robustness and generalizability of the relationship between education and INC. It underscores the importance of promoting and expanding educational opportunities for women in Ethiopia to empower them and provide them with the knowledge and resources needed to make informed decisions about their reproductive health.

The study findings uncovered a notable association between age groups and the average ideal number of children among women. Particularly, the analysis revealed that women in the age groups of 25–29, 30–34, 35–39, 40–44, and 45–49 exhibited a significantly higher average INC compared to younger women. This finding aligns with the results reported in the previous study by [25, 26], which supports the notion of age as a determinant of women’s desired number of children. The finding of the study revealed that the INC for women using contraceptives was significantly less than for non-user women. The result is in line with studies in [2, 27]. Women who were using contraceptives had a lower likelihood of having their expected number of children compared to women who were not using contraceptives. This result indicates that contraceptive use is associated with a reduction in fertility desires and preferences among women in Ethiopia. This finding is consistent with previous studies such as [28, 29], which also reported a similar relationship between contraceptive use and desired fertility. These studies consistently demonstrate that women who use contraceptives tend to have smaller family sizes or express a desire for fewer children compared to non-users.

This study is conducted based on cross-sectional data and hence not assessed the INC over time. The retrospective analysis causes inevitable bias and no external data sets were used for validation. Besides, other socioeconomic, demographic, biological, and behavioral characteristics were not considered. Thus, we authors would like to recommend that future researchers considered these characteristics as they might affect INC. Overall, studies on the ideal number of children can inform evidence-based policymaking, enabling policymakers to develop targeted interventions, allocate resources effectively, and shape policies that promote reproductive health, population well-being, and sustainable development.

## Conclusion

The spatial analysis revealed a significant clustering of the ideal number of children in the Ethiopia zone. Specifically, higher INC was observed in the Somali region, specifically in the Afder, Shabelle, Korahe, and Doolo zones. Among the various factors considered, women’s age, region, place of residence, women’s education level, contraception use, religion, marital status, family size, and age at first birth year were identified as significant predictors of the ideal number of children. These findings indicate that these factors play a crucial role in shaping reproductive preferences and decisions among women in the study population. Based on these findings, responsible bodies should prioritize targeted interventions and policies in high-risk regions to address women’s specific reproductive needs.

## Data Availability

The study used a released survey dataset that is available without participants’ identities. This was accessed based on a publicly available dataset that is freely available at http://dhsprogram.com/data/dataset/Ethiopia_StandardDHS_2016.cfm?fag=0.
